# Transition of Care Practices from Emergency Department to Inpatient: Survey Data and Development of Algorithm

**DOI:** 10.5811/westjem.2016.9.31004

**Published:** 2016-11-08

**Authors:** Sangil Lee, Jaime Jordan, H. Gene Hern, Chad Kessler, Susan Promes, Sarah Krzyzaniak, Fiona Gallahue, Ted Stettner, Jeffrey Druck

**Affiliations:** * The University of Iowa Carver College of Medicine, Department of Emergency Medicine, Iowa City, Iowa; † Harbor-UCLA Medical Center, Department of Emergency Medicine, Torrance, California; ‡ Alameda Health System, Highland Hospital, Department of Emergency Medicine, Oakland, California; § Duke University, Department of Emergency Medicine and Internal Medicine, Durham, North Carolina; ¶ Pennsylvania State University, Department of Emergency Medicine, State College, Pennsylvania; || University of Illinois at Peoria, Department of Emergency Medicine, Peoria, Illinois; # University of Washington, Department of Emergency Medicine, Seattle, Washington; ** Emory University, Department of Emergency Medicine, Atlanta, Georgia; †† University of Colorado, Department of Emergency Medicine, Aurora, Colorado

## Abstract

**Introduction:**

We aimed to assess the current scope of handoff education and practice among resident physicians in academic centers and to propose a standardized handoff algorithm for the transition of care from the emergency department (ED) to an inpatient setting.

**Methods:**

This was a cross-sectional survey targeted at the program directors, associate or assistant program directors, and faculty members of emergency medicine (EM) residency programs in the United States (U.S.). The web-based survey was distributed to potential subjects through a listserv. A panel of experts used a modified Delphi approach to develop a standardized algorithm for ED to inpatient handoff.

**Results:**

121 of 172 programs responded to the survey for an overall response rate of 70.3%. Our survey showed that most EM programs in the U.S. have some form of handoff training, and the majority of them occur either during orientation or in the clinical setting. The handoff structure from ED to inpatient is not well standardized, and in those places with a formalized handoff system, over 70% of residents do not uniformly follow it. Approximately half of responding programs felt that their current handoff system was safe and effective. About half of the programs did not formally assess the handoff proficiency of trainees. Handoffs most commonly take place over the phone, though respondents disagree about the ideal place for a handoff to occur, with nearly equivalent responses between programs favoring the bedside over the phone or face-to-face on a computer. Approximately two-thirds of responding programs reported that their residents were competent in performing ED to inpatient handoffs. Based on this survey and on the review of the literature, we developed a five-step algorithm for the transition of care from the ED to the inpatient setting.

**Conclusion:**

Our results identified the current trends of education and practice in transitions of care, from the ED to the inpatient setting in U.S. academic medical centers. An algorithm, which guides this process, is proposed to address the current gap in the standardized approach to ED to inpatient handoffs that were identified in the survey’s assessment of needs.

## INTRODUCTION

The handoff was defined as “the exchange between health professionals of information about a patient accompanying either a transfer of control over or of responsibility for the patient.”^1^ Patient handoffs were found to be responsible for medical errors and harmful to the patient, and the Institute of Medicine’s report, “To Err is Human,” highlighted handoffs as a potential area of improvement.^2^ The Joint Commission and the Accreditation Council for Graduate Medical Education (ACGME) recommended that sponsoring programs ensure and monitor an effective and structured handoff process. 3,4,5

Several studies reported the current practice of transition of care within the emergency department (ED), including previous studies by the Council of Residency Directors (CORD). 6 7 8 The CORD survey showed that over half of the respondents from academic EDs indicated that their EDs use a standardized handoff. 9 However, it is not known how emergency medicine (EM) residency programs are providing training around care transitions from the ED to inpatient settings.

The authors aimed to assess the current scope of handoff education and practices among resident physicians and to propose a standardized handoff algorithm to improve the transition of care from the ED to the inpatient setting.

## METHODS

### Survey Content

The authors conducted a cross-sectional survey targeted at EM residency programs in the United States. The survey was developed to address the initial two steps of the Kern model for medical curriculum development: 1) problem identification and general needs assessment; and 2) needs assessment for targeted learners.^10^ Content experts created a web-based survey to assess the current handoff practice from the ED to inpatient providers (Appendix 1. Survey questions).

### Survey Administration

We piloted surveys among the CORD Transition of Care (TOC) task force members and revised them before final administration. The survey was designed using the SurveyMonkey® platform (SurveyMonkey Inc., Palo Alto, California, USA. www.surveymonkey.com) and distributed to all members through the CORD listserv. The validity of using the CORD listserv as sample population has been described elsewhere.^7^,^11^,^12^ The responses were collected, and duplicated responses were removed and compiled for data analysis.

### Transition of Care Algorithm

Given the identified needs and opportunities in the transition of care, authors performed a review of the literature (Appendix 2. Search strategy). We used a modified Delphi technique to develop an algorithmic approach to conducting efficient handoffs from the ED to the inpatient setting, which served as a primer for the following two steps of the six-step Kern model: 3) goals and objectives, and 4) educational strategies.^10,13^ The algorithm was initially derived from the CORD TOC EM to EM handoff by Kessler et al.^7^ and implemented based on the literature review.^5^,^7^,^8^,^14^–^29^ The algorithm was modified and approved by seven experts.

### Statistical Analysis

Sample size calculations demonstrated that of the 172 programs surveyed as the true target population, 121 responses would give a 95% confidence interval with a 5% margin of error. We reported data using descriptive statistics and analyzed them by a two-sample test of proportion or Fisher’s exact test, as appropriate. We completed statistical analysis with JMP®, Version <10.0> (SAS Institute Inc., Cary, NC), and we reported p-values.

This study was declared exempt by the Alameda Health System.

## RESULTS

### Response Rate

A survey response was obtained from 121 out of 172 programs with the overall response rate being 70.3%.

### Transition of Care Curriculum, Handoff Structure and Safety Perception

Most programs offer handoff training to their resident physicians (Table 1). The type of training varied, with the most common form being instruction in the clinical setting, followed by handoff training during orientation, structured workshop/classes, educational packets or guides, and other methods. Less than half of the programs responded that they have a structured formal handoff process, yet the compliance among residents was variable. About half of responding programs responded that their current handoff system was safe and effective (Table 1).

### Handoff Assessment

Nearly half of responding programs stated that they do not formally assess handoff proficiency in resident physicians (Table 2). Otherwise, Table 2 shows the types of formal assessment methods of handoff proficiency in trainees.

### Current mode of Handoff and Recommended Handoff

Eighty-nine programs responded to the question of which mode of handoff process was used, and a handoff via phone was most common (Figure). On the other hand, of the 116 programs that responded to where the formal handoff should occur, answers were variable (Figure).

### Handoff Competency Assessment

Lastly, two-thirds of programs responded that their residents were extremely competent to competent in giving ED to inpatient handoffs (extremely competent 8/121, 6.6%; competent 71/121, 58.7%; somewhat competent, 41/121, 33.9%; incompetent, 1/121, 0.8%). There was a statistically significant association between achieving competency and instruction offered by attending or senior resident at clinical setting (p=0.006), but not with the handoff training during initial orientation (p=0.23), structured workshop (p=0.12), or educational packet (p=0.5).

### Handoff Algorithm

Given the identified need for handoff education and existing literature, authors developed a handoff algorithm ‘Prep-4Cs.’^8^,^15^–^18^,^30^ The handoff algorithm consists of five steps (Table 3).

## DISCUSSION

### Statement of Principal Findings

Our survey showed that most EM programs in the U.S. have some form of handoff training, the majority of them occurring during clinical setting. However, the handoff structure from ED to inpatient is neither well standardized nor followed. Only half of responding programs felt that their current handoff system was safe and effective. About half of the programs did not have a formal assessment. Handoffs most commonly take place over the phone, though respondents disagreed about the ideal place for a handoff to occur.

### Interpretation of Results Compared to Other Studies

The majority of EM programs in the U.S. now have some form of handoff training, which is in compliance with the ACGME common program requirement.^3^ The overall rate of the handoff education has now increased to 94% from 13% in 2013, reflecting the successful dissemination of handoff education.^7^ Hern et al. surveyed the trend of EM providers and concluded that there is an insufficient level of mandatory handoff training with varying results.^9^ Our study supports this finding and implies a further need for an effective handoff education.

This study demonstrates that handoff practice from ED to inpatient is not standardized, and even in places where a formal system exists, the compliance rate is not high. This is consistent with the existing literature, which showed that less than half of EM programs had a standardized handoff practice in 2013.^12^ A standardized handoff practice has been introduced to several inter-unit handoff processes, namely using mnemonics and checklists.^14^,^31^ A recent study showed that the use of communication training, mnemonics, and handoff structures decreased medical error in the pediatric inpatient setting.^32,33^ It implies that the introduction of standard mnemonics can be a starting point, yet programs may have to expand their curriculum into a handoff bundle tailored for ED to inpatient transition of care.

Only approximately half of the responding programs felt that their current handoff system was safe and effective, and about half of programs reported using a formal evaluation process for trainee proficiency. The existing literature identified a knowledge gap and the potential benefit of evaluation tools.^34,35^ It is prudent to develop validated evaluation tools to accurately assess the effectiveness and safety of handoffs.

About half of the respondents reported that the handoff occurred over the phone, yet there was no consensus on what mode of handoff would be ideal (Figure). A previous survey study demonstrated that ED to ED end of shift handoffs should ideally occur at the patient bedside, although many found that the handoffs actually occurred at the computer station.^7^ The most effective and safest practice model needs to be elucidated.

Lastly, while approximately two-thirds of programs reported that their residents were competent, this still leaves room for improvement either in training or assessment. Our analysis showed that only the presence of handoff training during clinical setting was associated with competency. Currently, there is no universally accepted competency assessment.^36^ As the program requirement includes the milestones for resident education, the level of competency needs to be accurately evaluated.

### Proposed Handoff Algorithm

The proposed algorithm “Prep-4Cs” (Table 3) is meant to provide some standardization while still allowing flexibility so institutions/programs ensure that their unique needs are met. Some institutions may already use a handoff mnemonic or template that can be incorporated into this algorithm. Prospective validation of this algorithm is required.

## LIMITATIONS

The study has several limitations. First, the response was based on each responder’s perception of the transition of care. Second, construct underrepresentation and construct-irrelevant variance could have affected the validity of the survey questions.^37^ Third, rater and recall bias need to be considered in the results, as the responder was anonymous in the survey.

## CONCLUSION

This study identified current trends of transitions of care from the ED to inpatient settings among academic medical centers in the U.S. and developed an algorithm to provide a foundation and springboard for educational strategies.

## Supplementary Information





## Figures and Tables

**Figure:**
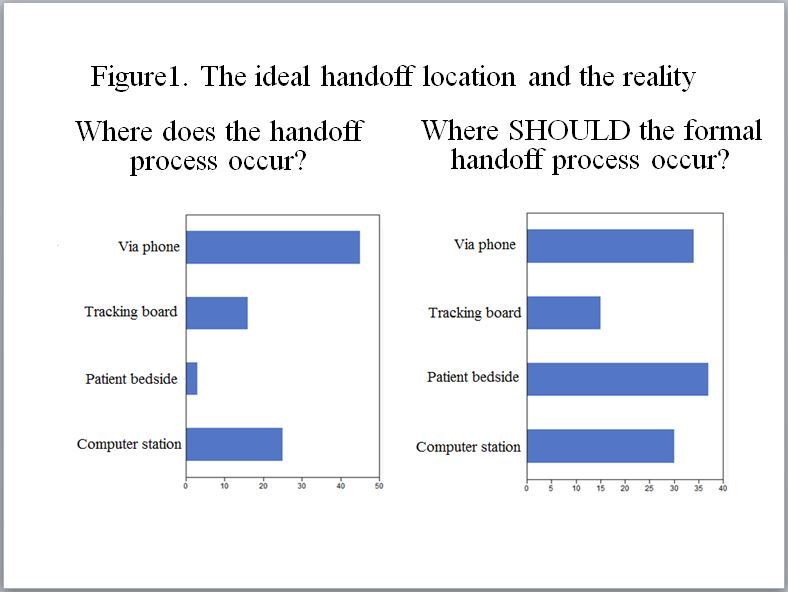
The ideal handoff location and the reality.

**Table 1:** Transition of care curriculum, handoff structure, and safety perception in emergency medicine training programs.

Response choices	Response rate/total, (%)*
Transition of care curriculum	
Attendings or senior residents provide handoff instruction in the clinical environment	90/121 (74.4)
Handoff training offered during the initial orientation	87/121 (71.9)
Structured workshop/classes to teach proper handoff procedure	27/121 (22.3)
Educational packets or guides for handoff	14/121 (11.6)
Other methods (simulation, policy and online instructions)	7/121 (5.8)
Handoff structure	
Structured handoff for ED to inpatient providers in place	45/119 (37.2)
How often do residents use a structured handoff?	
Always	9/45 (20)
Usually	13/45 (29)
Sometimes	19/45 (42)
Rarely	3/45 (6.7)
Safety perception	
Current handoff process is:	
Extremely safe and effective	2/121 (1.7)
Safe and effective	57/121 (47.1)
Somewhat safe and effective	56/121 (42.3)
Not safe or effective	6/121 (5.0)

* Multiple choices were allowed.

**Table 2:** Do you formally assess the handoff proficiency of your residents? If yes, how?

Response choices	Response rate/total, (%)
No, I do not formally assess the handoff of the residents.	59/121 (48.8)
Yes, assessment is done through scheduled one-on-one discussion with each resident.	7/121 (5.8)
Yes, assessment is done through regular written feedback/evaluation from EM personnel.	31/121 (25.6)
Yes, I ask the senior EM residents to assess the handoff proficiency of the junior residents.	15/121 (12.4)
Yes, residents/faculty from other services provide informal feedback on the quality of admission handoffs.	26/121 (21.5)
Yes, residents/faculty from other services provide regular formalized feedback on the quality of admission handoff.	3/121 (2.5)
Other methods	16/121 (13.2)

**Table 3:** EM-IM transition of care algorithm “PREP-4Cs.”

PREP-4Cs
Step 1. Preparation Immediate access to patient information, assessment, access to images, labs and medical record. Time commitment (2–5min) Space with minimal interruption
Step 2. Contact Sender and receiver identify themselves, including name and service “Face to face or voice to voice” to share real time information
Step 3. Communicate patient information Structured sign-out format for each institution Recommended as feasible mnemonics (alphabetical order) for EM-IM transition, cited from Riesenberg table14: HANDOFFS (Hospital location, Allergies, Name, DNR, Ongoing problem, Fact about hospitalization, Follow up, Scenarios) I PASS (Introduction, Patient name, Assessment, Situation, Safety concerns) SBAR (Situation, Background, Assessment, Recommendation) SBARR (Situation, Background, Assessment, Recommendation, Read back) SHARQ (Situation, History, Assessment, Recommendation, Questions) SIGNOUT (Sick, Identifying data, General hospital course, New events, Overall health status, Upcoming possibilities, Tasks) SOAP (Subjective, Objective, Assessment, Plan) Identification of high-risk patient: if high risk, explain the following: Why they are high risk How they may decompensate Planning for continued care Frequency of reassessment Code status or POLST
Step 4. Closing the loop Invitation for asking questions Discuss pending tests, treatment and delegate clear delineation of responsibility on follow ups Receiver verification of information
Step 5. Conclusion Conclusion Documentation of the transition of care Documentation of plan Open invitation for re-contact and discussion if a future need arises
