# Supplementary material for: Transition of Care Practices from Emergency Department to Inpatient: Survey Data and Development of Algorithm
Source: West J Emerg Med. 2016 Nov 8;18(1):86–92. doi: 10.5811/westjem.2016.9.31004 (PMC5226771; doi:10.5811/westjem.2016.9.31004)
Supplement: Supplementary file 1 [file wjem-18-86-s001.pdf]

Exit this survey

2015 Emergency Medicine Provider to INPATIENT Handoff

This survey is designed to assess current ED provider to INPATIENT provider handoff practices and also to determine where possible learning deficiencies may exist. If you hold a position at multiple hospitals/sites, please answer questions based on experiences at your primary hospital/site. Completion of this survey is voluntary. On Behalf of the CORD Transition of Care Task Force, thank you for your time.

*** 1. Which program are you from.**

List of Programs - (By State)

Program Name [ ]

2. Please indicate any type of ED to INPATIENT handoff training that is offered to residents/students in your program. (Check all that apply)

- ☐ No training is offered
- ☐ Handoff training during initial orientation to the program
- ☐ Structured workshops/classes to teach proper handoff procedure
- ☐ Instruction by attending/senior resident within the clinical environment
- ☐ Distributed educational packets/guides

Other (please specify)

[ ]

3. Do you currently use a structured and standardized handoff system in the for ED Provider to INPATIENT Provider (resident to resident) transitions in care?

- ○ Yes
- ○ No

Other (please specify)

[ ]

4. Please rate how safe/effective you think your current handoff system is at providing all necessary information to the incoming provider or team.

	Not safe/effective	Somewhat safe/effective	Safe/effective	Extremely safe/effective
Safety/effectiveness rating	○	○	○	○

5. If you have a formalized system, how often do your residents follow the formal handoff procedure/system?

	Rarely (less than 25% of the time)	Sometimes (about 50% of the time)	Usually (about 75% of the time)	Almost always (about 100% of the time)
My residents follow the procedure...	○	○	○	○

6. Do you formally assess the handoff proficiency of your residents? If yes, how? (check all that apply)

- ☐ No, I do NOT formally assess the handoff proficiency of the residents.
- ☐ Yes, assessment is done through scheduled one-on-one discussions with each resident.
- ☐ Yes, assessment is done through regular written feedback/evaluation from EM personnel.
- ☐ Yes, I ask the senior EM residents to assess the handoff proficiency of the junior residents.
- ☐ Yes, Residents/faculty from other services provide informal feedback on the quality of admission hand-offs
- ☐ Yes, Residents/faculty from other services provide regular formalized feedback on the quality of admission hand-offs

I use another method of assessing the resident's handoff proficiency (please describe below).

7. Do you use any of the following tools to assist in the handoff process? (check all that apply)

- ☐ No tools are used
- ☐ Written template or other written aids
- ☐ Mnemonics
- ☐ Computer/Electronic Sign-out

Please describe your mnemonic or template

8. If you use a tool, please rate your level of satisfaction with the tool.

	Unsatisfied	Somewhat satisfied	Satisfied	Extremely satisfied
Please indicate your level of satisfaction with the tool:	○	○	○	○

9. If your residents do use a formal handoff process, where does the handoff process occur? (please select one answer only)

- ○ Patient bed-side (face to face)
- ○ Individual computer stations (face to face)
- ○ Patient Tracking Board (face to face)
- ○ Via Phone

Other (please specify)

10. In your opinion, where SHOULD the formal handoff process occur? (please select one answer only)

- Patient bed-side (face to face)
- Individual computer stations (face to face)
- Patient Tracking Board (face to face)
- Via Phone

Other (please specify)

11. As a standard of practice, are handoffs (notes) documented in the respective patient's record?

- Yes
- No

Other (please specify)

12. Please rate your residents' competency with giving ED to INPATIENT handoffs.

- ED Incompetent
- Somewhat competent
- Competent
- Extremely competent

13. Please provide any other feedback on this survey or on transitions of care in general. Thanks

Done
