# Supplementary material for: Transition of Care Practices from Emergency Department to Inpatient: Survey Data and Development of Algorithm
Source: West J Emerg Med. 2016 Nov 8;18(1):86–92. doi: 10.5811/westjem.2016.9.31004 (PMC5226771; doi:10.5811/westjem.2016.9.31004)
Supplement: Supplementary file 2 [file wjem-18-86-s002.docx]

Earliest results is 1976.

Cochrane CENTRAL yields 41. Without changing strategy, EMBASE yields 1158 (including meeting abstracts from 2010 to present)

| **Ovid MEDLINE(R) In-Process & Other Non-Indexed Citations and Ovid MEDLINE(R)** 1946 to Present | | | |
| --- | --- | --- | --- |
| **#** | **Searches** | **Results** | **Search Type** |
| 1 | ((hand* adj2 (off* or over*)) or handoff* or handover* or sbar or "shift report*" or "intershift report*" or (sign* adj2 (out* or off* or over*)) or signoff* or signover* or signout*).mp. [mp=title, abstract, original title, name of substance word, subject heading word, keyword heading word, protocol supplementary concept word, rare disease supplementary concept word, unique identifier] | 78743 | Advanced |
| 2 | 1 or patient handoff/ | 78743 | Advanced |
| 3 | ((patient* or inpatient*) adj3 (transition* or transfer*)).mp. [mp=title, abstract, original title, name of substance word, subject heading word, keyword heading word, protocol supplementary concept word, rare disease supplementary concept word, unique identifier] | 17385 | Advanced |
| 4 | ((intra or between or within) adj2 (department* or ward* or unit* or service* or shift*)).mp. or exp hospital departments/ [mp=title, abstract, original title, name of substance word, subject heading word, keyword heading word, protocol supplementary concept word, rare disease supplementary concept word, unique identifier] | 174556 | Advanced |
| 5 | (((intra or "in") adj hospital) or inhospital* or interservice* or interdepartment*).mp. [mp=title, abstract, original title, name of substance word, subject heading word, keyword heading word, protocol supplementary concept word, rare disease supplementary concept word, unique identifier] | 57395 | Advanced |
| 6 | interprofessional relations/ or continuity of patient care/ or interdisciplinary communications/ or information transfer/ or information dissemination/ or patient care planning/ or clinical competence/ or communicat*.mp. [mp=title, abstract, original title, name of substance word, subject heading word, keyword heading word, protocol supplementary concept word, rare disease supplementary concept word, unique identifier] | 423823 | Advanced |
| 7 | (tool* or intervention* or checklist* or "check list*" or sheet* or aid*1 or protocol* or standardi* or skills or training* or teach* or curricul* or "minimum data*" or procedur* or "quality improve*").mp. [mp=title, abstract, original title, name of substance word, subject heading word, keyword heading word, protocol supplementary concept word, rare disease supplementary concept word, unique identifier] | 3038361 | Advanced |
| 8 | outcome*.mp. or patient safety/ or "safety".mp. or delay*.mp. or "time to treatment".mp. or "sentinel event*".mp. or "adverse event*".mp. or "length of stay".mp. or LOS.mp. or mortality.mp. or malpractice*.mp. or safety management/ or "near miss".mp. or missed.mp. or reported.mp. or faulty.mp. or breakdown*.mp. or preventable.mp. or exp medical errors/ or "situational awareness".mp. [mp=title, abstract, original title, name of substance word, subject heading word, keyword heading word, protocol supplementary concept word, rare disease supplementary concept word, unique identifier] | 3642596 | Advanced |
| 9 | comparative study/ or qualitative*.mp. or qualitative studies/ or interview*.mp. or questionnaire*.mp. or focus group/ or observational*.mp. or cohort*.mp. or prospective*.mp. or retrospective*.mp. or randomized controlled trial.pt. or randomi*.mp. or randomized controlled trial as topic/ or cross-sectional study/ or case-controlled trial.pt. [mp=title, abstract, original title, name of substance word, subject heading word, keyword heading word, protocol supplementary concept word, rare disease supplementary concept word, unique identifier] | 3870270 | Advanced |
| 10 | ("mixed method*" or observation*).mp. [mp=title, abstract, original title, name of substance word, subject heading word, keyword heading word, protocol supplementary concept word, rare disease supplementary concept word, unique identifier] | 629690 | Advanced |
| 11 | 2 and (3 or 4 or 5) and (6 or team*.mp.) [mp=title, abstract, original title, name of substance word, subject heading word, keyword heading word, protocol supplementary concept word, rare disease supplementary concept word, unique identifier] | 530 | Advanced |
| 12 | 2 and 8 | 29481 | Advanced |
| 13 | 12 and (3 or 4 or 5 or 6 or team*.mp.) [mp=title, abstract, original title, name of substance word, subject heading word, keyword heading word, protocol supplementary concept word, rare disease supplementary concept word, unique identifier] | 2601 | Advanced |
| 14 | limit 13 to (clinical trial, all or clinical trial, phase i or clinical trial, phase ii or clinical trial, phase iii or clinical trial, phase iv or clinical trial or comparative study or controlled clinical trial or evaluation studies or meta analysis or multicenter study or observational study or pragmatic clinical trial or randomized controlled trial or "review" or validation studies) | 914 | Advanced |
| 15 | (11 or 13) and program evaluation/ | 89 | Advanced |
| 16 | (11 or 14) and (9 or 10) | 862 | Advanced |
| 17 | (7 or 8) and (11 or 14) | 1250 | Advanced |
| 18 | 15 or 16 or 17 | 1304 | Advanced |
| 19 | 18 not (letter or editorial).pt. | 1298 | Advanced |
| 20 | remove duplicates from 19 | 1239 |  |
